# Clinical spectrum of tuberculous optic neuropathy

**DOI:** 10.1007/s12348-012-0079-5

**Published:** 2012-05-22

**Authors:** Ellen J. Davis, Sivakumar R. Rathinam, Annabelle A. Okada, Sharon L. Tow, Harry Petrushkin, Elizabeth M. Graham, Soon-Phaik Chee, Yan Guex-Crosier, Eva Jakob, Ilknur Tugal-Tutkun, Emmett T. Cunningham, Jacqueline A. Leavitt, Ahmad M. Mansour, Kevin L. Winthrop, William L. Hills, Justine R. Smith

**Affiliations:** 1 Casey Eye Institute, Oregon Health & Science University, 3375 SW Terwilliger Boulevard, Portland, OR 97239 USA; 2 Department of Medicine, Oregon Health & Science University, Portland, OR USA; 3 Department of Public Health and Preventive Medicine, Oregon Health & Science University, Portland, OR USA; 4 Department of Cell and Developmental Biology, Oregon Health & Science University, Portland, OR USA; 5 Department of Ophthalmology, Aravind Eye Hospital, Madurai, India; 6 Department of Ophthalmology, Kyorin University School of Medicine, Tokyo, Japan; 7 Department of Ophthalmology, St. Thomas Hospital, London, UK; 8 Singapore National Eye Centre, Singapore, Singapore; 9 Jules Gonin Eye Hospital, University of Lausanne, Lausanne, Switzerland; 10 Interdisciplinary Uveitis Center, University of Heidelberg, Heidelberg, Germany; 11 Department of Ophthalmology, Istanbul University, Istanbul, Turkey; 12 Department of Ophthalmology, California Pacific Medical Center, San Francisco, CA USA; 13 Department of Ophthalmology, Stanford University, Palo Alto, CA USA; 14 Department of Ophthalmology, Mayo Clinic, Rochester, MN USA; 15 Department of Ophthalmology, American University of Beirut, Beirut, Lebanon

**Keywords:** Optic neuropathy, Tuberculosis, Presentation, Visual outcome

## Abstract

**Purpose:**

Tuberculous optic neuropathy may follow infection with *Mycobacterium tuberculosis* or administration of the bacille Calmette–Guerin. However, this condition is not well described in the ophthalmic literature.

**Methods:**

Ophthalmologists, identified through professional electronic networks or previous publications, collected standardized clinical data relating to 62 eyes of 49 patients who they had managed with tuberculous optic neuropathy.

**Results:**

Tuberculous optic neuropathy was most commonly manifested as papillitis (51.6 %), neuroretinitis (14.5 %), and optic nerve tubercle (11.3 %). Uveitis was an additional ocular morbidity in 88.7 % of eyes. In 36.7 % of patients, extraocular tuberculosis was present. The majority of patients (69.4 %) had resided in and/or traveled to an endemic area. Although initial visual acuity was 20/50 or worse in 62.9 % of 62 eyes, 76.7 % of 60 eyes followed for a median of 12 months achieved visual acuities of 20/40 or better. Visual field defects were reported for 46.8 % of eyes, but these defects recovered in 63.2 % of 19 eyes with follow-up.

**Conclusion:**

Visual recovery from tuberculous optic neuropathy is common, if the diagnosis is recognized and appropriate treatment is instituted. A tuberculous etiology should be considered when evaluating optic neuropathy in persons from endemic areas.

## Introduction

Tuberculous optic neuropathy includes multiple potential optic nerve involvements that may follow infection with *Mycobacterium tuberculosis* or vaccination or therapeutic administration of the bacille Calmette–Guerin (i.e., attenuated *Mycobacterium bovis*). After antimicrobial agents against *M. tuberculosis* became available approximately 70 years ago, patient survival improved and reports of clinical manifestations of tuberculous eye disease increased in the literature [1]. As summarized in the comprehensive review of intraocular tuberculosis by Gupta et al. [2], unrelated case reports have described various optic neuropathies in patients with tuberculosis, including neuroretinitis, papilledema, papillitis, optic neuritis, retrobulbar neuritis, and optic nerve tubercle; disease may reflect direct infection or an associated hypersensitivity reaction. Published series of tuberculous optic neuropathy, however, have totaled to less than ten patients and/or been limited to patients with tuberculous meningitis.

To address the lack of information regarding the spectrum of tuberculous optic neuropathy that may present to the ophthalmologist, a group of 13 neuro-ophthalmologists and inflammatory eye disease specialists retrospectively collected specified clinical data on 49 patients. The resultant data set provides a description of types of optic nerve involvement and associated eye disease, patient demographics, presenting symptoms, methods of diagnosis, modes of treatment, and visual outcomes.

## Methods

Ophthalmologists who had managed cases of tuberculous optic neuropathy were identified through postings on the electronic communities of the American Uveitis Society, the International Uveitis Study Group, the Association of Proctor Fellows, and the North American Neuro-Ophthalmology Society. In addition, authors of recently published cases of tuberculous optic neuropathy were found through literature searches using Ovid MEDLINE. Individuals were invited to complete a data collection sheet that was approved by the Oregon Health & Science University Institutional Review Board for their patients with tuberculous optic neuropathy.

Contributing ophthalmologists were asked to submit the following information: patient characteristics including gender, age at presentation, ethnicity, countries of residence and travel, and risk factors for contracting tuberculosis; optic nerve involvement(s) (using the diagnoses listed by Gupta et al. [2], i.e., optic nerve tubercle, papilledema, papillitis, optic neuritis, retrobulbar neuritis, neuroretinitis, or other involvements); other tuberculous ocular disease and systemic manifestations; presenting symptom(s); duration of follow-up; visual acuity at presentation and at final visit; and complications, including visual field defects and optic atrophy. If any subject had also suffered from uveitis or scleritis, description of the uveitis according to SUN Working Group criteria [3] or classification of the scleritis per Watson and Hayreh [4] was required.

Criteria used to determine tuberculosis as the cause of the optic neuropathy were requested, following the recommendations of Gupta et al. [2] which were updated to include the interferon-gamma (IFN-γ) release assay as an alternative to the Mantoux skin test, per current guidelines [5]. These criteria include: consistent ocular signs; positive Mantoux reaction or IFN-γ release assay; active or old tuberculous lesion on chest imaging; polymerase chain reaction (PCR) detection of *M. tuberculosis* DNA in ocular fluid samples; identification of acid-fast bacilli by microscopy or culture of ocular or other tissue samples; and/or positive response to four-drug anti-tuberculosis treatment (i.e., isoniazid, rifampicin, ethambutol and pyrazinamide). Information regarding investigations undertaken to exclude other etiologies for the optic neuropathy, as well as optic nerve or brain imaging studies, was also collected. In addition, the pharmacological approach was required.

## Results

Clinical data were obtained from 13 providers at 11 ophthalmology clinics in 9 different countries relating to 49 patients and 62 eyes with a diagnosis of tuberculosis associated optic neuropathy. Seven cases were previously published as individual reports [6, 7] or in a case series [8]. The cohort included 26 males (53 %) and 23 females (47 %), with a median age at presentation of 36 years (range = 13–76 years) (Table 1). The racial backgrounds of patients were diverse, including Asian (*n* = 32, 65.3 %), White (*n* = 10, 20.4 %), and Black (*n* = 7, 14.3 %). Continents of residence and/or travel of these individuals included Asia (*n* = 44, 89.8 %), Europe (*n* = 14, 28.6 %), Africa (*n* = 8, 16.3 %), and North America (*n* = 8, 16.3 %); some individuals resided in or traveled to more than one continent. The most common presenting ocular symptoms were decreased vision (*n* = 42, 85.7 %) and ocular pain (*n* = 13, 26.5 %), but some patients had other complaints, including visual field defects, ptosis, altered color vision, diplopia, ocular redness, and floaters (*n* = 1–3, 2–6 %).

**Table 1:** Characteristics of the study sample (*n* = 49 persons)

Variable	Number of patients (%) or median (range)
Gender	
•Male	26 (53 %)
•Female	23 (47 %)
Age (years)	36 (13-76)
Laterality	
•Unilateral	36 (73.5 %)
•Bilateral	13 (26.5 %)
Presenting symptoms	
•Decreased vision	42 (85.7 %)
•Pain (includes ocular pain, facial pain, headache)	13 (26.5 %)
•Visual field loss	3 (6.1 %)
•Ptosis	3 (6.1 %)
•Color vision loss	2 (4.1 %)
•Diplopia	2 (4.1 %)
•Ocular injection	2 (4.1 %)
•Floaters	2 (4.1 %)
•Other^a^	7 (14.3 %)
Race	
•Asian	32 (65.3 %)
•White	10 (20.4 %)
•Black	7 (14.3 %)
Continents of residence or travel	
•North America	8 (16.3)
•Europe	14 (28.6)
•Africa	8 (16.3)
•Asia	44 (89.8)

^a^Other: epiphora (*n* = 1), eyelid edema (*n* = 1), facial numbness (*n* = 1), nasal congestion (*n* = 1), photophobia (*n* = 1), photopsia (*n* = 1), transient visual obscuration (*n* = 1)

Following the description of potential optic nerve involvements given by Gupta et al. [2], optic nerve involvement in 62 eyes of 49 patients was classified as: papillitis in 32 eyes (51.6 %), neuroretinitis in 9 eyes (14.5 %), optic nerve tubercle in 7 eyes (11.3 %), retrobulbar neuritis in 5 eyes (8.1 %), and optic neuritis in 5 eyes (8.1 %) (Table 2). Other optic nerve involvements, not included in this description, were: compressive optic neuropathy in five eyes (8.1 %) and anterior ischemic optic neuropathy in two eyes (3.2 %). Other ocular involvements included: uveitis in 55 of 62 eyes (88.7 %) and 37 of 49 patients (75.5 %), orbital apex syndrome in 8 eyes (12.9 %) of 8 patients (16.3 %), posterior scleritis in 1 eye (1.6 %) of 1 patient (2.0 %), stromal keratitis in 1 eye (1.6 %) of 1 patient (2.0 %), and central retinal vein occlusion in 1 eye (1.6 %) of 1 patient (2.0 %). Other ocular involvements were observed both in eyes affected with optic neuropathy and in unaffected eyes. Per SUN criteria, of the 55 eyes with uveitis: 1 eye had anterior uveitis (1.8 %), 3 eyes had intermediate uveitis (5.5 %), 34 eyes had posterior uveitis (61.8 %), and 17 eyes had panuveitis (30.9 %). Onset of uveitis was sudden in 29 (52.7 %) and insidious in 26 (47.3 %) eyes. Duration of uveitis was limited in 22 (40.0 %), persistent in 31 (56.4 %), and unspecified in 2 (3.6 %) eyes. The course of uveitis was acute in 21 (38.2 %), recurrent in 1 (1.8 %), chronic in 29 (52.7 %), and unspecified in 4 (7.3 %) eyes.

**Table 2:** Optic nerve and other ocular involvements (n = 62 eyes)

	Number of eyes (%)
Optic nerve involvements^a^	
•Papillitis	32 (51.6 %)
•Neuroretinitis	9 (14.5 %)
•Optic nerve tubercle	7 (11.3 %)
•Compressive optic neuropathy	5 (8.1 %)
•Retrobulbar neuritis	5 (8.1 %)
•Optic neuritis	5 (8.1 %)
•Anterior ischemic optic neuropathy	2 (3.2 %)
•Papilledema	0 (0 %)
Other ocular involvements^a^	
•Uveitis	55 (88.7 %)
•Orbital apex syndrome	8 (12.9 %)
•Posterior scleritis	1 (1.6 %)
•Stromal keratitis	1 (1.6 %)
•Central retinal vein occlusion	1 (1.6 %)
Description of uveitis (*n* = 55 eyes)	
Location	
•Anterior	1 (1.8 %)
•Intermediate	3 (5.5 %)
•Posterior	34 (61.8 %)
•Panuveitis	17 (30.9 %)
Onset	
•Sudden	29 (52.7 %)
•Insidious	26 (47.3 %)
Duration	
•Limited	22 (40.0 %)
•Persistent	31 (56.4 %)
•Unspecified	2 (3.6 %)
Course	
•Acute	21 (38.2 %)
•Recurrent	1 (1.8 %)
•Chronic	29 (52.7 %)
•Unspecified	4 (7.3 %)

^a^Multiple optic nerve or other ocular involvements were observed in some patients

Criteria used to diagnose tuberculous optic neuropathy included: consistent clinical signs (*n* = 49, 100 %); positive Mantoux reaction (*n* = 38, 77.6 %); positive IFN-γ release assay (*n* = 10, 20.4 %); tuberculous lesion on chest imaging (*n* = 4, 8.2 %); identification of acid-fast bacilli by microscopy or culture of extraocular tissue samples (*n* = 3, 6.1 %); and/or positive response to antituberculosis treatment (*n* = 44, 89.8 %), although in some cases such treatment was not “four-drug”. Of the 49 patients, 32 individuals had disease that was contiguous with the optic nerve, including uveitis, posterior scleritis, orbital mass, and optic nerve tuberculoma. Of the 17 patients without such lesions, 10 individuals had uveitis and 4 individuals showed evidence of extraocular tuberculosis. Of the remaining 3 patients, 1 patient was a previously published case of tuberculous orbital apex syndrome, [8] and 2 patients underwent testing for other potential causes of the optic neuropathy. Applying the guidelines for diagnosis of intraocular tuberculosis that were defined by Gupta et al. [2] modified as described in the “Methods”, all 49 cases were classified as “presumed” tuberculous optic neuropathy. Risk factors for tuberculosis in the study group were: residence in and/or travel to an endemic area (http://www.who.int/tb/publications/global_report/2007/xls/global.xls.) (*n* = 34, 69.4 %), personal or family history of tuberculosis (*n* = 9, 18.4 %), medical conditions associated with susceptibility (i.e., diabetes mellitus, *n* = 5, 10.2 % and malignancy, *n* = 1, 2.1 %), and health care profession (*n* = 1, 2.1 %). Involvement of extraocular tissues or organs outside the eye was reported in 18 of 49 persons (36.7 %), including lung (*n* = 8, 16.3 %), meninges (*n* = 4, 8.2 %), lymph node (*n* = 4, 8.2 %), bone (*n* = 1, 2 %), and kidney (*n* = 1, 2 %). These data are listed in Table 3.

**Table 3:** Diagnosis, systemic involvement and risk factors for tuberculosis (*n* = 49 persons)

Variable	Number of patients (%)
Criteria used for diagnosis of tuberculosis	
•Consistent ocular signs	49 (100 %)
•Positive response to antituberculous treatment	44 (89.8 %)
•Positive Mantoux reaction	38 (77.6 %)
•Positive IFN-gamma release assay	10 (20.4 %)
•Active or old lesion(s) consistent with pulmonary tuberculosis on chest imaging	4 (8.2 %)
•Identification of acid-fast bacilli by microscopy or culture	
•Extraocular	3 (6.1 %)
•Intraocular	0 (0 %)
•Positive *M. tuberculosis* PCR from ocular fluid	0 (0 %)
Systemic involvements	
•Pulmonary	8 (16.3 %)
•Central nervous system: meningeal	4 (8.2 %)
•Lymphatic	4 (8.2 %)
•Other^a^	2 (4.1 %)
•None	31 (63.3 %)
Risk factors	
•Lived in or travel to an endemic area	34 (69.4 %)
•Personal or family history of tuberculosis	9 (18.4 %)
•Diabetes mellitus	5 (10.2 %)
•Health care worker	1 (2.1 %)
•Malignancy	1 (2.1 %)

^a^Other systemic involvements: bone (*n* = 1); renal (*n* = 1)

The use of optic nerve or brain computerized axial tomography and magnetic resonance imaging, and the selection of investigations to exclude other etiologies for the optic neuropathy, varied between cases. Overall, optic nerve or brain imaging was performed in 19 patients (38.8 %). The differential diagnosis of tuberculous optic neuropathy depends on multiple factors that include the specific optic involvement, the presence and type of other ocular pathology, and geography. In addition, financial considerations may impact which tests are ordered for a specific patient. Thus, the approach to excluding other diagnoses for this series of patients was necessarily heterogeneous, and consequently, it was not possible to generate one standard algorithm for the approach to the differential diagnosis of the optic neuropathy. Taking into account the type of optic nerve involvement, and detailed clinical history and examination findings, alternative etiologies that were considered by the providers included other infections (i.e., syphilis, toxoplasmosis, cat scratch disease, Lyme disease, leptospirosis, systemic fungal infection, HIV-associated disease, and other forms of infectious meningitis); systemic inflammatory diseases (i.e., multiple sclerosis, neuromyelitis optica, sarcoidosis, Vogt–Koyanagi–Harada syndrome, Behçet’s disease, and other systemic vasculitidies); ocular inflammatory conditions (i.e., uveitis and posterior scleritis); and vascular, neoplastic, toxic, and hereditary forms of optic neuropathy.

Of the 49 patients, 47 individuals received antituberculosis treatment (Table 4). One patient was lost to follow-up prior to treatment, and another patient refused treatment. Management was with four-drug antituberculosis treatment in 29 patients (59.2 %) and with other antibiotic regimens in 18 patients (36.7 %). In addition to antibiotics, 28 patients (57.1 %) were treated with oral prednisone, with stated maximum daily doses varying between 20 and 120 mg. One patient received intravenous methylprednisolone in addition to oral prednisone. Two other patients were given sub-Tenon’s corticosteroid injections; one of these patients also took oral prednisone.

**Table 4:** Treatment regimens (*n* = 49 patients)

Treatment	Number of patients (%)
Antibiotic alone	18 (36.7 %)
Corticosteroid alone	0 (0 %)
Antibiotic + corticosteroid	29 (59.2 %)
None^a^	2 (4.1 %)
Antibiotic	
•Four-drug regimen^b^	29 (59.2 %)
•Other antituberculosis treatment	18 (36.7 %)
Corticosteroid	
•Oral	28 (57.1 %)
•Intravenous	1 (2.0 %)
•Local	2 (4.1 %)

^a^None: lost to follow-up (*n* = 1); refused antituberculous treatment (*n* = 1)

^b^Four-drug regimen: isoniazid + rifampicin + pyrazinamide + ethambutol

Snellen visual acuity was reported at presentation and, for 47 patients who were followed for a median of 12 months (range = 1–81 months), at final visit (Table 5). For 62 eyes, initial visual acuity was 20/40 or better in 23 eyes (37.1 %), 20/50 or worse in 39 eyes (62.9 %), and 20/200 or worse in 24 eyes (38.7 %). At final examination, 46 of 60 eyes (76.7 %) achieved visual acuities of 20/40 or better, while 14 eyes (23.3 %) retained visual acuities of 20/50 or worse and 6 eyes (10.0 %) retained visual acuities of 20/200 or worse. There was no difference in achieving a visual acuity of 20/40 or better for eyes treated with corticosteroid (69.7 %) versus those not so treated (85.2 %) (*p* > 0.05, Fisher’s exact test, two-tailed). Of the 5 persons who either did not receive treatment or did not have a positive response to treatment, 2 patients were lost to follow-up. For the 3 affected eyes of the 3 remaining patients, visual acuity improved at least two lines in 2 eyes and dropped at least two lines in 1 eye. Numbers in individual groups were too small for making meaningful correlations between optic nerve involvement and visual acuity outcome. However, a two-line improvement in visual acuity was reported for eyes with each form of optic nerve involvement, with the exception of anterior ischemic optic neuropathy. Visual field defects were documented in 29 eyes (45.2 %). The most common defects were enlarged blind spot (*n* = 8, 27.6 %) and central scotoma (*n* = 4, 13.8 %), but 12 different defects were reported. The course of the visual field change was known for 19 of these eyes: 6 eyes (31.6 %) experienced full recovery, 6 eyes (31.6 %) experienced partial recovery, and 7 eyes (36.8 %) sustained permanent visual field defects. Thirteen eyes (20.1 %) had evidence of optic atrophy on examination.

**Table 5:** Snellen visual acuity and complications (*n* = 62 eyes)

	Number of eyes (%)	
Snellen visual acuity		
	Initial (*n* = 62 eyes)	Final (*n* = 60 eyes)
>20/50	23 (37.1 %)	46 (76.7 %)
≤20/50	39 (62.9 %)	14 (23.3 %)
≤ 20/200	24 (38.7 %)	6 (10.0 %)

Complications		

Visual field defects	29 (46.8 %)	
Defects (*n* = 29 eyes)		
•Enlarged blind spot	8 (27.6 %)	
•Central scotoma	4 (13.8 %)	
•Altitudinal defect	2 (6.9 %)	
•Peripheral defect	2 (6.9 %)	
•Enlarged blind spot + paracentral scotoma	2 (6.9 %)	
•Central scotoma + peripheral defect	2 (6.9 %)	
•Other^a^	6 (20.7 %)	
•Not specified	3 (10.3 %)	
Recovery (*n* = 29 eyes)		
•Full	6 (20.7 %)	
•Partial	6 (20.7 %)	
•No	7 (24.1 %)	
•Not specified	10 (34.5 %)	
Optic atrophy	13 (21.0 %)	

^a^Other: enlarged blind spot + central scotoma (*n* = 1), cecocentral scotoma + peripheral defect (*n* = 1), paracentral scotoma (*n* = 1), altitudinal defect + peripheral defect (*n* = 1), quadrantanopia (*n* = 1), 3-quadrantanopia (*n* = 1)

## Discussion

Renewed interest in ocular tuberculosis has been evident in the ophthalmic literature over the past decade [2, 9, 10]. This attention is explained by factors that include: increasing prevalence of tuberculosis worldwide, recognition of *M. tuberculosis* as the etiological agent in specific subtypes of uveitis, introduction of new diagnostic tools for the infection, and emergence of drug-resistant tuberculosis. Published studies have focused on uveitis, which is the most common ocular manifestation of the infection. We sought to contribute to the literature on ocular tuberculosis by describing the clinical spectrum of optic nerve involvement.

In our series of 49 cases of tuberculous optic neuropathy, the disease was typically unilateral, and the majority of patients presented with painless loss of vision. More than two thirds (69.4 %) of the affected individuals had resided in and/or traveled to an endemic area for the infection. Nerve involvements were diverse, but in approximately one half of patients took the form of papillitis. Approximately 90 % of eyes had uveitis, which in over 90 % of the cases was posterior uveitis or panuveitis. Extraocular tuberculosis—particularly pulmonary and meningeal—was present in 36.7 % of patients. The vast majority of patients received treatment with antituberculosis antibiotics, and over one half were also treated with corticosteroid. Visual outcomes were generally good. At final review, 76.7 % of eyes achieved visual acuities of 20/40 or better, and only 10.0 % of eyes had visual acuities of 20/200 or worse. In addition, among the 19 of 29 eyes that developed visual field defects and for which follow-up was available, 63.2 % experienced complete or partial recovery.

There are no diagnostic criteria designated for tuberculous optic neuropathy specifically, and the diagnosis of ocular tuberculosis in general continues to pose great difficulty to ophthalmologists. Problems relate to the limitations of all available tests, as well as the obvious difficulty in taking a biopsy of affected intraocular tissues. These challenges were recently summarized in a comprehensive review by Vasconcelos-Santos et al. [11] and further illustrated with the series of clinicopathologically correlated cases reported by Wroblewski et al. [12]. The situation has improved over the past 10 years, with the successful commercialization of ocular fluid analysis for bacterial DNA by the polymerase chain reaction [13, 14] and tests of peripheral blood lymphocyte reactivity to *M. bacterium*-specific antigens [15–17]. However, while significant concerns remain, multiple different diagnostic criteria are currently in use. We followed the recommendations of Gupta et al. [2] which can be summarized as: consistent clinical signs; and positive results of ocular investigations or positive results of systemic investigations or therapeutic response to antituberculosis treatment; and exclusion of other potential causes of uveitis. Using this set of criteria, the clinician may define “confirmed” (i.e., with positive results of ocular investigations) and “presumed” cases. In the vast majority of cases [18], as also exemplified by our series, the diagnosis of ocular tuberculosis is presumed. Other diagnostic guidelines are more stringent, but also are more difficult to use in practice due to the acknowledged difficulty in obtaining ocular samples for testing [10, 19].

Different treatment regimens have been described for the management of ocular tuberculosis, as reviewed by Gupta et al. [2]. Recommendations published in 2003 by the US Centers for Disease Control are that treatment of tuberculosis should be with the combination of isoniazid, rifampicin, pyrazinamide, and ethambutol [20]. A recent analysis of the published literature suggests treatment of ocular tuberculosis may vary from these guidelines at approximately half of ophthalmology clinics around the world [21]. Four-drug antituberculosis treatment was given in 59.2 % of our patients, but all patients who agreed to treatment and/or continued in follow-up received antimicrobial treatment. We would like to highlight the importance of collaborating with an infectious disease physician when managing tuberculous optic neuropathy, as has been emphasized by others [2, 10, 11]. One unanswered question is: are patients with tuberculous optic neuropathy treated with ethambutol and/or isoniazid at increased risk of optic nerve toxicity, given an already compromised optic nerve status? We did not observe medication-induced optic neuropathy among the 31 patients who received ethambutol plus isoniazid or the additional 16 patients who received isoniazid, but not ethambutol. However, we recommend patients be monitored closely for this complication. Deterioration in color vision, visual acuity, and/or visual field following initial improvement may indicate drug toxicity. In addition, pyridoxine is routinely combined with isoniazid to protect against peripheral neuropathy.

Systemic corticosteroid is frequently added to antimicrobial treatment of intraocular tuberculosis, although practice patterns are not established [2, 10, 11]. Use of periocular corticosteroid injections is also reported [18, 22]. Fifty-nine percent of our patients received systemic or local corticosteroid as part of their treatment. This was always done concurrently with antituberculosis therapy; it is clear that uveitis occurring in the context of latent or manifest tuberculosis is significantly more likely to recur when treated with corticosteroid alone [23]. We observed no significant difference in numbers achieving 20/40 or better visual acuity for patients treated with corticosteroid versus those not so treated, however. Interestingly, in their series of 157 patients with suspected tuberculous uveitis, Ang et al. [17] also noted no significant difference in outcomes between patients treated with antituberculosis drugs combined with corticosteroid therapy versus antituberculosis drugs alone. Despite these results, we recognize the potential value of corticosteroid in reducing inflammation in selected cases of ocular tuberculosis, as has been widely reported [2, 9, 10]. We recommend caution, however, when considering periocular injection of long-acting corticosteroid, as the duration of action may extend beyond the course of antituberculosis treatment.

Limitations of our study include the retrospective nature of data collection, ascertainment bias, and the difficulties of studying a disease for which diagnostic criteria are not established. On the other hand, our study represents a large series of patients of tuberculous optic neuropathy, and standardized data collection has allowed us to summarize optic nerve and other ocular involvements, presenting clinical features, diagnostic and treatment approaches, and visual acuity and visual field outcomes. The practical implications of our study include the need for a high index of suspicion for tuberculosis when evaluating optic neuropathy in patients who have resided in or traveled to an endemic area. Prior treatment of tuberculosis does not preclude the possibility of recurrent infectious disease [24]. In 2010, the Centers for Disease Control published their recommendation that either a tuberculin skin test or an IFN-γ release assay be used in the routine diagnosis of *M. tuberculosis* infection [5]. Our study also highlights the importance of investigating for extraocular involvement, particularly pulmonary or meningeal, when optic nerve disease is present. Finally, a collaborative effort by the ophthalmologist and an infectious disease physician in developing an antituberculosis treatment schedule can result in a good visual outcome for patients with tuberculous optic neuropathy.
